# Elusive implementation: an ethnographic study of intersectoral policymaking for health

**DOI:** 10.1186/s12913-018-2864-9

**Published:** 2018-01-30

**Authors:** Ditte Heering Holt, Morten Hulvej Rod, Susanne Boch Waldorff, Tine Tjørnhøj-Thomsen

**Affiliations:** 1 University of Southern Denmark, National Institute of Public Health, Studiestræde 6, Copenhagen, 1455 Denmark; 2 Metropolitan University College, National Research Centre for Disadvantaged Children and Young People, Kronprinsesse Sofies Vej 35, 2000 Frederiksberg, Denmark; 3 Copenhagen Business School, Department of Organization, Kilen, Kilevej 14a, 2000 Frederiksberg, Denmark

**Keywords:** Intersectoral policymaking, Intersectoral collaboration, Health in all policies, Policy process, Implementation, Municipal health promotion, Local government

## Abstract

**Background:**

For more than 30 years policy action across sectors has been celebrated as a necessary and viable way to affect the social factors impacting on health. In particular intersectoral action on the social determinants of health is considered necessary to address social inequalities in health. However, despite growing support for intersectoral policymaking, implementation remains a challenge. Critics argue that public health has remained naïve about the policy process and a better understanding is needed. Based on ethnographic data, this paper conducts an in-depth analysis of a local process of intersectoral policymaking in order to gain a better understanding of the challenges posed by implementation. To help conceptualize the process, we apply the theoretical perspective of organizational neo-institutionalism, in particular the concepts of rationalized myth and decoupling.

**Methods:**

On the basis of an explorative study among ten Danish municipalities, we conducted an ethnographic study of the development of a municipal-wide implementation strategy for the intersectoral health policy of a medium-sized municipality. The main data sources consist of ethnographic field notes from participant observation and interview transcripts.

**Results:**

By providing detailed contextual description, we show how an apparent failure to move from policy to action is played out by the ongoing production of abstract rhetoric and vague plans. We find that idealization of universal intersectoralism, inconsistent demands, and doubts about economic outcomes challenge the notion of implementation as moving from rhetoric to action.

**Conclusion:**

We argue that the ‘myth’ of intersectoralism may be instrumental in avoiding the specification of action to implement the policy, and that the policy instead serves as a way to display and support good intentions and hereby continue the process. On this basis we expand the discussion on implementation challenges regarding intersectoral policymaking for health.

**Electronic supplementary material:**

The online version of this article (10.1186/s12913-018-2864-9) contains supplementary material, which is available to authorized users.

## Background

The public health officers in ‘Townville’ sit around the table in their meeting room and discuss how to conduct the “*kickoff meeting*” launching the municipality’s new health policy. The policy has just been adopted by the City Council. Now the administration must develop an intersectoral implementation strategy. The public health officers agree that it is very important that the policy aims are comprehensible and “*by all means not fluffy*” to non-health departments. They want to ensure support among all departments and not least to produce a high quality strategy. To achieve this purpose an external consultant, who helps plan the process, asks the public health officers to define what the policy aims mean. He asks about the aim “*healthy measures*”. They struggle to come up with answers. There is silence. One suggests that it is “*the structural*”. Another disagrees. She thinks it should not be limited to “*regulation*”. They discuss whether “*structural*” is more than “*regulation*”. They do not want regulation, as this is not considered politically feasible with the center-right majority in the City Council. The consultant suggests it is about “*working systematically with the framework conditions*”. They all agree. They are happy with how he phrases it. One asks him to repeat it so they can write it down. Several of them make notes. There is a sense of relief among them. They move on to talk about the timeline of the process.

This excerpt highlights the key question of our paper: how can we understand the way good intentions to turn policy rhetoric into intersectoral action for health resulted in a process of producing abstract rhetoric and vague plans? The paper adopts an ethnographic perspective on the process of intersectoral policymaking for health in a Danish municipality. We investigate the apparent paradox that ideas about intersectoral policymaking have become popular among politicians and public health professionals, while simultaneously experienced as a great challenge to implement in practice [1–3].

For more than 30 years, policy action across sectors has been celebrated as a necessary and viable way to affect the social factors impacting on health [4]. In 1986, the Ottawa Charter [5] emphasized the importance of healthy public policy. More recently, it has been followed by calls for health in all policies and joined-up government to act on the social determinants of health and hereby ensure better population health and health equity [3, 6, 7]. While many researchers, politicians and public health professionals agree on this intent, implementation of intersectoral policy remains a challenge [3, 8, 9].

Within public health, implementation is often conceptualized as the process of turning policy rhetoric into action [10, 11]. This is based on the assumption that policy will guide action by allocating resources and providing guidance on the division of responsibilities, as well as setting goals and targets to be met [9, 12, 13]. However, Ollila finds that “[i]deas [are] more easily transferred into rhetoric than practice, and implementation of intersectoral health policies remains challenging” [10]. While political science has long known that it is challenging to move from statements of intent to implementation [14–16], critics find that public health has remained naïve about the policy process and has paid little attention to how it affects implementation [17]. Generally, researchers call for a better understanding in public health of the processes and mechanisms involved with intersectoral policymaking [8, 12, 17–20].

The aim of this paper is to provide a better understanding of the process and social dynamics of intersectoral policy implementation. First we present our theoretical framework and then the methodological approach. In the Results we first give a detailed description of the implementation process to illustrate how an apparent failure to implement policy was effected by the reproduction of abstract rhetoric and vague plans. In the subsequent section we analyze the challenges and argue that idealization of universal intersectoralism, doubts about economic outcomes, and inconsistent demands about decision-making functioned to decouple the intersectoral strategy from directing action. In the Discussion we expand on the implementation challenges and discuss the implications of the study in relation to the existing research. We argue that decoupling rhetoric from action may not necessarily constitute a failure of implementation in the traditional sense, as the process serves to display good intentions, maintains high values that would otherwise be rejected, and keeps the process running.

### Theoretical framework

The study is informed by organizational neo-institutionalism, and applies the concepts of rationalized myth and decoupling [21], as they hold explanatory power relating to the challenge of turning rhetoric into action.

Organizational neo-institutionalism is defined by a focus on how organizations take in institutionalized reform ideas as part of organizational rhetoric, because these ideas have become popular in the institutional environment, even ‘taken-for-granted’, as the legitimate and efficient way of organizing [21, 22]. These institutionalized reform ideas are conceptualized as rationalized myths. Rationalized myths excite and grab attention as powerful solutions to organizational challenges, due to their appearance as effective instruments, not their efficiency as instruments for change [21]. A rationalized myth, however, may disturb the organization’s daily operations because it ensures legitimacy but not necessarily efficiency. Thus decoupling may be the organizational response to cope with rationalized myths as hypothesized by Meyer and Rowan [21]. The concept of decoupling suggests that reform ideas are adopted in rhetoric and policies as ‘window-dressing’ without affecting daily operations [23]. By decoupling formal rhetoric from organizational action, organizations are able to carry out their core tasks and cope with many, often opposing, demands from the institutional environment.

## Methods

This study is part of an explorative study investigating intersectoral efforts for health in ten Danish municipalities [24]. In this paper we focus on a single municipality referred to as ‘Townville’. The first author, DHH, followed the intersectoral process of implementing a municipal-wide health policy during a period of 1 year from August 2013 to August 2014. This was from when a new intersectoral health policy was about to be adopted and the following process of developing an intersectoral implementation strategy and establishing intersectoral governance mechanisms. Participant observation, together with semi-structured and informal interviews, were the main methods of data production to provide an in-depth ethnographic account of the process of local intersectoral policymaking for health [25–27].

Townville is an exemplary case in the sense that it represents general challenges and aspirations we found in the overall study. Moreover, Townville offered a unique opportunity to study intersectoral policymaking, because intersectoral collaboration was a key priority and great efforts were invested to achieve it. The case thus enables us to learn about critical aspects of intersectoral policymaking, which are most often not accessible for researchers to study directly. To gain access, DHH approached a public health officer who organized initial interviews. A meeting was set up with the Public Health Office (PHO) and the Children and Youth Secretariat to formalize an agreement outlining the aim and extent of the fieldwork. DHH was then invited when the strategy was on the agenda. The presence and aim of the research was briefly introduced at all intersectoral meetings, and oral permission was always asked to record discussions. The study was approved by the Danish Data Protection Agency.

### The field

Several health determinants are related to the local governmental level [28], and municipalities are often considered to possess features which place them in a key position to address population health due to their local governance models and responsibilities in a number of sectors [29, 30]. Denmark is a universalistic welfare state [31] with rather decentralized decision-making [32]. The main healthcare services such as hospitals and general practitioners are within the regional jurisdiction. A local government reform in 2007 made health promotion and prevention the responsibility of municipalities [33, 34]. This was to a great extent based on ideas about intersectoral action for health, as municipalities were expected to possess great opportunities to integrate health within local welfare services, such as schools, employment service, local planning, and social services among others [35]. Thus Danish municipalities provide a great opportunity to examine intersectoral policymaking and action at the local level.

Townville is a medium-sized municipality with an urban center, surrounding villages and agricultural hinterland with a population of around 70,000. Since the 2007-reform, Townville had initiated various health promotion and prevention interventions. Townville experienced rising costs related to non-communicable diseases and changing demographics of an aging population, as well as social inequalities in health. To address these concerns the new health policy was intended to establish broader, intersectoral commitment, and a more strategic approach to public health.

**Fig. 1:**
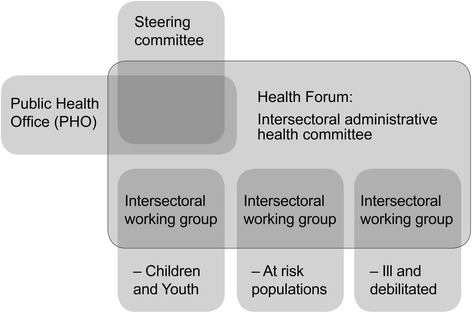
Organization of the intersectoral process adapted from a visualization produced by PHO

The intersectoral process was organized in intersectoral meeting groups; 1) a steering committee; 2) an intersectoral health committee referred to as Health Forum; and 3) three intersectoral working groups divided according to three main target groups: children and youth, at risk populations, and sick and debilitated. The process was planned by a project group in Townsville’s PHO. The public health officers functioned as coordinators and facilitated all intersectoral meetings. Figure 1 provides an overview of the intersectoral organization as it was visualized by PHO.

### The fieldwork

DHH participated as participating observer [36] and followed the work of the steering committee, the Health Forum, the intersectoral working group representing Children and Youth Services – referred to as the working group – as well as PHO (see Table 1). It is noteworthy that politicians did not play a central part in the process and generally seemed to support the political proposals prepared by the administration on this issue. The fieldwork thus focused on the intersectoral collaboration of the administration in order to convey the greatest insight regarding the intersectoral process and included the few political meetings where the implementation strategy was on their agenda. Analyzing the role of the local politicians in more detail is beyond the scope of this paper, but see Holt [37] for further discussion on this matter.

**Table 1:** Overview of the meetings DHH participated in

Type of meeting	Number of meetings
*Internal meetings in PHO*	19
*Intersectoral working group: 1 kick off meeting, 1 “pitch workshop”, and 2 (out of 3) working group meetings*	4
*Health Forum: Intersectoral administrative health committee – approximately 25 mid-level managers*	2
*Steering committee – top-level management*	3
*Political meetings*	City Council meeting: 1 Political committee meeting: 1
*Other intersectoral meetings (management seminar in Children and Youth Services + informal meeting on intersectoral collaboration between PHO and Children and Youth Services)*	2
Total number of meetings attended during the fieldwork	32

Meetings are a common way to structure and restructure social life in contemporary organizations [38], and thus provide a unique opportunity to study organizational negotiations about what intersectoral policymaking for health is and should be, according to participants. Beyond the formal meetings, ‘hanging out’ prior to and after meetings and informal situations during breaks constituted part of the fieldwork. These situations provided opportunities for informal interviews and conversations that helped to establish trust, and contextualize and unfold observations from the meetings. The observations were initiated and followed up by semi-structured interviews with key participants representing top-level management, public health officers and involved civil servants mainly from Children and Youth Services. Eleven scheduled interviews in total (see Table 2 for an overview, and Additional file 1 for a template of the interview guide).

**Table 2:** Overview of scheduled semi-structured interviews

Interviews, August 2013	
*Municipal director*	
*Head of school department (Children and Youth Department)*	
*Head of specialized services (Children and Youth Department)*	
*Executive secretary (Children and Youth Department)*	
*Head of Employment Services*	
*Manager of public health office (PHO)*	
*Public health officer (PHO)*	
Follow up interviews, August 2014	
*Municipal director*	
*School principal*	
*Manager of public health office (PHO)*	
*Public health officer (PHO)*	

### Analytical strategy and analysis

The empirical material consists of field notes, sound recordings of meetings and interview transcripts and summaries, as well as organizational documents like internal meeting reports and power-point slides, and formal documents like adopted strategies and policies. DHH has translated quotes and edited them for readability. Participants’ intentions are described as they were depicted in meetings.

The study employed an abductive research strategy [39], which is characterized by producing theoretical hunches for unexpected research findings – such as our observation that the overall good intentions in Townville did not lead to the expected action. These hunches are then developed in an iterative process of working with the empirical data in ‘dialogue’ with the theoretical literature [39, 40]. We analyzed the intersectoral process by repeated readings of the empirical data to write up situations and practices related to intersectoral collaboration and the process of policy implementation. We used holistic data organization [41], where explanations are derived from analysis of the ‘whole’ process. This provides an understanding of the “intricately interwoven” parts of the data set relating to the complex intersectoral process [41]. To follow the principles of abductive analysis, the concepts of rationalized myth and decoupling, as well as health promotion literature on barriers and facilitators of intersectoral policymaking informed the analysis.

## Results

The Results consists of two main sections, each organized in three subsections. First, we give a detailed description of the ethnographic case. Next, we provide an analysis of the implementation challenges by applying the concepts of rationalized myth and decoupling.

### The process of developing an intersectoral implementation strategy

In this section we give a detailed chronological description of the intersectoral process. This is structured to present 1) the participants’ intentions, 2) the example of the “*pitch*” template, and 3) the outcome in terms of the approved strategy. We use the example of the pitch to illustrate how the process reproduced abstract rhetoric rather than plans for action.

#### High hopes and strong beliefs

“*Everyone talks about breaking down the silos, but we try to achieve it*”.

Participants in Townville described that they were in a process of breaking down organizational silos. This was expressed in phrases like “*breaking down the silos*”, “*breaking the columns*” or as “*creating coherence and consistency*”. Intersectoriality was a main priority of the process and great efforts were put into achieving this: The policy had been developed in an intersectoral process and was accompanied by intersectoral governance mechanisms such as Health Forum and the working groups, intersectoral political consultations and a public conference, a public hearing, dedicated funding to support new initiatives, as well as commitment and leadership from top-level management, and a political mandate for intersectoral collaboration. Overall the process enjoyed great support among participants, who generally showed great enthusiasm. For instance, after the launch of Health Forum, several participants came over to congratulate the public health manager and told how excited they were. Only on one occasion did a local manager question whether it was necessary to introduce a new strategy. He did not receive much support from his colleagues though. Instead they argued for the necessity of an intersectoral strategy to ensure coordinated efforts:

“*You can’t save the world with an intervention in your department alone. It needs to be consistent with what is done in social services, in employment services etc. This is why we need to have a strategy.*”

The belief in intersectoralism was paralleled by a belief in control by strategic planning. At numerous occasions participants expressed how it was essential that the policy, and particularly the strategy, would set priorities and give direction for action: “*It must be a tool to make priorities. It must give a direction*”. Generally, participants believed that introducing the intersectoral strategy developed through a thorough intersectoral process, would help control action across sectors, thereby creating coherence and consistency.

#### Structuring the strategy: The pitch template

“*[The health policy] has been approved, but that in itself does not produce health. So now it must be brought to life […] We cannot bring the policy to life alone in our small office. It requires that everyone […] must be involved*”.

With these words the public health manager introduced the working groups to their task. The quote sums up their main intentions for the process: to move from policy to practice and to engage the entire municipality in the policy implementation. Implementation came across as a shared ambition that was highly desired, but constituted great challenges. For instance the working group initially described one of their aims as: “*to do what we say we do*” and “*Townville employees must live up to our own policies*”. Another example was a discussion on local policies:

“*The thing that’s so damned about all the policies we have, because we have a billion policies and strategies. […] we can’t say that it is not described. Everything is described. It is a matter of whether it is being done.*”

There was a general frustration among many participants who wanted a closer connection between policies and action.

To ensure this connection and achieve implementation, the project group introduced the “*pitch template”*. The “*pitch*” was a table on one sheet of paper with fixed phrases to fill out in order to present interventions concisely. It was presented as an “*innovation tool*”, as the template should help clarify and convey ideas without any “*woolly talk*”, thus ‘pitching’ them to get clout:

“*Academic and administrative language sneaks in too easily. All this must be stripped away. We must communicate clearly. If we can’t communicate our ideas clearly we can’t collaborate.*”

The pitch was conceptualized as a tool to establish collaboration and moving interventions from idea to action. On this background it was introduced to structure the strategy.

However, working with the pitch was difficult for participants, who struggled to concretize their ideas. For instance, during the workshop participants kept changing the overall theme of the pitch as they struggled to fill in the blanks in the template. E.g. relating to the aim mental health, they discussed whether preventing suicide or promoting general wellbeing was the objective. They were not sure what constituted the greatest problems or best line of action. Moreover, whenever participants were forced by the templates to make ideas explicit, these were the rare occasions when the atmosphere changed and became tense or tired.

Another challenge the pitch highlighted was the sheer number of objectives in the policy. The health policy outlined three overall aims: “*healthy measures*”, “*mental health*”, and “*equality in health*”. Each aim included numerous explicit objectives, as well as statements of intent. The coordinators produced a table to create an overview, which amounted to 53 objectives. This included the objective to meet basic recommendations from the national prevention guidelines, which alone consisted of 176 recommendations. As such, the policy did not provide the direction they attributed to it and prioritization remained a key challenge. This was highlighted by the pitch template’s tight structure. Participants struggled to suggest specific action while maintaining the purpose of the strategy as an overarching document:

“*It’s a challenge now that the implementation strategy is still at a very strategic, general level. If we use pitch it will be at least 30 pitches for each target group, so we need to lump it together in associated themes*”.

As a consequence, over a period of some weeks PHO decided to use the pitch template to describe more general areas of intervention, and thus moved away from the original intention to communicate plans for action.

The working group produced five pitches describing overall areas of intervention in Children and Youth Services: “*health integrated as part of core operations*” which focused on professional competences; “*strengthening parenthood and mental health of young families*”; “*local health strategies in all daycare centers, schools and special services*”; “*promote health among the youth*”; and “*implement the national prevention guidelines*”. The pitches were distributed to the working group and members of Health Forum for final comments. Most feedback was positive but some did suggest a few changes, for instance:

A manager comments on “*strengthening parenthood*”. The pitch suggests (among other things) to add an extra visit by community nurses to mothers within the first year of birth. The manager questions whether the costs of adding an extra visit would correspond with an equivalent outcome of better health. The coordinator replies that this proposal is based on the decision to meet national recommendations, which endorse five visits within the first year. Townville only offers 4. She adds that she will change the wording of the pitch and concludes: “*I will try to rephrase this part of the pitch in relation to strengthening the parenting role, but without making a specific proposal*”.

The pitches were then adjusted to incorporate the last comments, thus rephrasing some details to make them less specific.

The draft of the strategy, including pitches from all three working groups, was then distributed among Health Forum and the Steering committee for approval. At this point, top-management in Children and Youth Services (where two of four top-managers were members of the steering committee) rejected the strategy. At a meeting between the top-management group of Children and Youth Services, the public health manager and working group coordinator, the pitches were rejected. DHH was not present at this meeting, but learned later that the pitches were still considered too explicit, despite the reworking. As a consequence it was finally decided to remove the pitch template completely from the implementation strategy. The strategy then consisted of general descriptions presenting the headlines and general aims of the 16 areas of intervention, but without explicating the action involved.

#### Approving the strategy

The strategy was then (verbally) presented to the City Council for discussion. Here some politicians reacted negatively to the long recitation of the now 16 suggested areas of intervention. The City Council did not express explicit opposition to the content, but advised that the number was cut down to create a better overview. PHO edited the strategy to make it more easily readable for the politicians. However, instead of removing suggestions they joined them together into six themes: “*child obesity*”; “*child and adolescent mental health*”; “*intersectoral substance use prevention*”; “*better health for vulnerable populations*”; “*well-being among sick, debilitated and at risk populations*”; and “*increased intersectoral collaboration regarding old-age medical patients*”. The suggested areas of intervention were listed in boxes underneath the themes as “*examples*”. As a result all suggested interventions were still potential future actions, although no action was prioritized and decided upon. Moreover, the suggestions remained highly abstract, only introduced by a heading. Therefore the final strategy did not provide the prioritization and direction for action, which was initially desired. The strategy was subsequently presented to the political committee with the mandate to approve health interventions, who adopted it without further changes.

In follow-up interviews, participants expressed general approval of the strategy and evaluated the process a success. For instance the director of Children and Youth Services concluded: “*It’s a good plan”*. He told that his employees had started referring to the health policy and talked a lot more about health than they used to. He believed this would contribute positively to the future implementation.

### Producing generalities to maintain good intentions

This case raises the analytical question of why the very explicit and dedicated attempt to produce a clear strategy to direct action resulted in vague plans and abstract rhetoric, and how this can be considered satisfactory by participants. In this section we analyze our case as an example of decoupling. We show how intersectoralism was idealized, while tensions between inconsistent demands were not resolved, but were maintained in abstract rhetoric and vague plans. We argue that the strategy served as a document of good intentions, while seemingly being decoupled from having any significant impact upon the operations of the municipal organization. We argue that the very ‘myth’ of intersectoralism was instrumental in avoiding the specification of action that was intended to implement the policy.

#### The myth of intersectoralism

Intersectoralism was never defined, but referred interchangeably to various meanings. Despite this, the benefits of intersectoral efforts were never questioned by participants who praised it as a means to produce “*coherence*” and “*consistency*”, and hereby “*generate synergies*”, despite hardly any analysis or detailed plan of how this would be achieved. Intuitively they all agreed that avoiding “*everyone running in opposite directions*” was essential, reflecting the taken-for-granted appeal of the rationalized myth [21].

Intersectoralism was idealized and often considered more valuable than action within a single sector. For instance, the fact that the Children and Youth Policy already had health related objectives was seen as a barrier to overcome rather than an advantage. On several occasions the project group commented on this as a challenge: “*it is particularly cumbersome with Children and Youth Services, because they have already started their own process. It would be easier if they were waiting around*”. In contrast, the ideal was the entire municipality functioning as one coordinated whole. E.g. it was often stressed that ensuring all citizens were met by the same approach was essential, and local pilot projects were not valued very highly if they were not systematically disseminated.

The steering committee briefly discussed whether the intersectoral process necessarily should include all departments, or whether some division e.g. between ‘child’ and ‘adult’ services could be made to ensure relevance while maintaining efficiency. However this was soon dismissed, as they believed Townville had a shared challenge that needed to be addressed intersectorally. A top-level manager said: “*drugs and alcohol, the disadvantaged, they are cross-cutting problems, and transition from child to adult services […] I believe it is dangerous to separate it. I’m afraid we won’t reach the synergy then*”. Attempts to designate certain areas for intersectoral collaboration were avoided in order to preserve the ideal of universal intersectoralism. Thus, the belief in a shared strategy to create coherence and consistency across all municipal departments functioned as a barrier to designate more specific action, as it entailed an unwillingness to move towards the local and unique. It appears that the greater the efforts to include and involve all departments, hence to do intersectoralism ‘right’, the more diluted (vague and abstract) the result, as the strategy only remained a shared document by maintaining the high level of abstraction. The idealization of universal intersectorialism thus legitimized and encouraged the reproduction of generalities. By keeping rhetoric and plans at an overall abstract level, Townville maintained the ‘myth’ of intersectoralism as a panacea; benefitting all aspects of the organization, being a universal solution everyone could support.

#### Economic expectations and doubts

Part of the myth of intersectoralism was expectations about economic benefits. The public health manager explained: “*It’s this idea that the gold is buried between the chairs*” – chairs referring to different services and/or legislations. He referred to Townville’s tight economy and planned budget cuts and explained that intersectoral collaboration was believed to initiate innovative solutions across departments. This was expected to result in improved efficiency and better effectiveness of municipal services. Generally, participants talked about “*investing in health*”, assuming that better health would reduce the demand for expensive services, hereby improving Townville’s economic situation. Effects were expected to be simultaneously health effects and economic effects, as expressed by the public health manager: “*we need to do what works, so we get maximum value for money […] when we talk about evidence, we refer to best practice, best value for money, health economics and evaluations.*”

Despite the powerful myth, doubts about outcomes also defined the process. Uncertainty about economic outcomes was particularly mentioned as a concern. An example is a discussion in the project group about expected economic outcomes:

They discuss whether to suggest “*reduced co-financing for hospitals*” as an outcome. One says that maybe it is not within their control. They do not know whether the regional hospitals will just admit more patients: “*co-financing is dangerous to add as effect*”. They decide that it is probably out of their control and agree it is better to change the wording to “*increased economic flexibility*”. They are pleased with how this does not tie them to deliver specific savings on co-financing but includes the derived savings expected from better health promotion and prevention efforts.

The excerpt illustrates how doubts about outcomes functioned to produce abstract rhetoric. Especially economic concerns seemed to impose an uncertainty that encouraged decoupling by making plans and rhetoric less explicit. For instance, when asked about the decision to remove the pitches from the strategy, the managing director explained that they had to make the strategy less specific because: “*the City Council won’t approve an implementation strategy that cost 100 million [DKK]. And I believe it could easily cost that. Everyone knows they won’t accept that.*” Despite small economic funding for new initiatives, it was a general assumption – sometimes expressed as an explicit requirement – that the policy was to be implemented within existing budgets. So regardless of expectations about economic benefits, the uncertainty about economic outcomes meant that Townville talked about investing in health, but adopted a strategy with no requirements for action, in order to satisfy budget demands.

#### Tensions regarding decision-making

Beyond economic expectations, the myth of intersectoralism was characterized by a strong intention to make the process inclusive and participatory. Organizing the process in multiple intersectoral groups was based on the assumption that an inclusive process would make it easier to implement the policy: partly because everyone had been involved in phrasing the challenges and suggesting solutions, which would thus produce a better strategy; partly because the inclusive process in itself would create ownership and commitment. As the public health manager noted: “*we should be facilitators […] not public health experts […] we know we won’t get anywhere if it is directed from the central […] so it is important to establish ownership and commitment […] It is so important to get it bottom-up*”. However, while aspiring for a role as facilitators, the project group was simultaneously concerned with ensuring the quality of the strategy. The public health officers saw their role as “*adding professionalism*” and “*equip participants*” to ensure that interventions were based on public health knowledge. They were determined not to let personal “*unprofessional*” conceptualizations of health among staff define local interventions. As a result the project group experienced a tension between the ideal of bottom-up involvement and their professional aspirations of ensuring public health knowledge as the basis of decision-making.

Another tension regarding decision-making was between demands for political leadership and management flexibility. The working group requested direction from their politicians: “*the problem is deciding what is good, what is good enough […] we need the politicians to make these decisions*”. The ambition to make a powerful strategy involved the demand for strong political leadership making priorities. However, this was countered by a simultaneous demand that politicians should not interfere in details of management: “*Townville is decentralized, so politicians shouldn’t be involved in the details. Politicians decide on the overall objectives but leave space for management.*” This concern was voiced by both top-management and participants in the working group. They did not want politicians to interfere in the details of operations. Hence, the process entailed conflicting demands regarding political leadership making both prioritizations and space for managerial control.

The process was therefore characterized by tensions regarding who should decide on priorities, and on what grounds. However these tensions were rarely articulated explicitly. Rather, the different demands remained abstract expressions of good intentions, and participants generally agreed to the various demands despite underlying inconsistencies. On few occasions, when inconsistencies between such demands were voiced explicitly e.g. relating to budget- or political requirements, abstract rhetoric was actively produced to avoid resistance, as the excerpts in the introduction and on the pitch “*strengthening parenthood*” illustrate. Abstract rhetoric accordingly functioned to maintain inconsistencies, making the strategy a document of good intentions. This way, the myth of intersectoralism remained intact, while the strategy was decoupled from having substantial impact on other parts of the municipal organization.

## Discussion

In this section we sum up our findings and discuss implications in relation to implementation of intersectoral policymaking.

We find that the myth of intersectoralism posed a barrier to the ambition of moving from overall statements of intent to more specific plans for action. The process produced activity, but activity that seemed somewhat parallel to and decoupled from daily operations. So despite elaborate governance mechanisms – which are often recommended as the means to foster intersectoral collaboration [9, 42, 43] – the process did not entail the expected move from rhetoric to action.

From existing literature on joined-up government and partnerships we know that boundary spanning skills are required in order to successfully manage intersectoral collaboration [44–47]. These skills include managerial creativity and flexibility in order to exploit collaborative opportunities, which Bardach refers to as craftsmanship [45]. Managing intersectoral collaboration requires a specific set of ‘soft power’ skills such as problem-solving skills, coordination skills (getting people to the table), brokering skills (seeing what needs to happen), flexibility, deep knowledge of the system, and a willingness to undertake the emotional labor associated with relational working ([46], p. 8). Both Carey and Crammond [46] and Hunter and Perkins [47] find that strong leadership at multiple levels are particularly important together with establishing trust and good working relationships between partners. Additionally, Hunter and Perkins [47] identify a number of barriers to efficient partnership working, among others: different agency priorities, lack of a shared goal that can create ownership, reluctance to share power, and missing links between levels. Applied to our case, we find that while the positive characteristics of trust, good working relationship and strong leadership were all present in Townville, so too were barriers such as different departmental and sectoral priorities and lack of a shared goal at the operational level. As such, the challenge of implementing the policy may be no surprise. The contribution of our analysis is to highlight how the myth of intersectoralism was instrumental in escaping the development of shared priorities and a common goal: The idealization of universal intersectoralism, together with uncertainty about economic outcomes and inconsistent demands regarding decision-making, meant that Townville maintained and produced abstract rhetoric and plans, and thus decoupled the implementation strategy from directing organizational action as no clear priorities were made. By not specifying plans for actions, Townville to a great extent maintained the myth of intersectoralism.

However the concept of decoupling suggests a rather strategic decision to talk and act to satisfy different demands. In our case, we find that maintaining and reproducing abstract rhetoric and plans was not only a matter of actively decoupling talk from action. Whereas economic concerns seemed to actively encourage decoupling, other tensions were not explicitly articulated to the same extent. For the most part, everyone seemed happy to maintain and reproduce the generalities. Thereby they maintained the tensions between inconsistent demands, thus making the strategy a document displaying and maintaining good intentions.

These findings may be explained by the Swedish organization theorist Nils Brunsson [48, 49] who convincingly argues that popular organizational models can be adopted “with much talk of beautiful principles and little discussion about practice” [49]. Brunsson finds that agreement is better achieved by keeping talk abstract and simple, as it is easier to agree on abstract terminology than more complex, precise and explicit plans [48]. However, the consequence being that the talk is unable to provide a good basis for joint action [48, 49]. Moreover, by producing abstract rhetoric and vague plans Townville ensured compatibility between the strategy and potential future actions. This may even be considered a way to avoid complete decoupling, because it allowed Townville the necessary flexibility to continue implementation. Paradoxically, the strong intentions to implement the policy might thus be the very reason that the strategy ended up being so washed out. As Townville strongly wished to turn the policy into action, they needed to leave enough flexibility for the strategy to accommodate inconsistent demands and function side by side with budget cuts, multiple national reforms and various traditions and organizational cultures in different sectors.

### Implementation

Our case therefore questions the notion of implementation as a matter of moving from policy rhetoric to action. Pinto et al. [50] along with Freiler et al. [12] define implementation of health in all policies (HiAP) as “actions to carry out governmental decisions as specified through legislation, formal strategy or mandate”. Thus, when formal talk – such as policies and strategies – cannot be made into action there is a problem of implementation. According to Brunsson [48], when talk cannot be realized in action this may be because the ideas are not suitable for translation because they are not clear-cut and precise enough, e.g. when strategies result in a compromise based on contradictory demands and expressed in vague terms [48].

A more general question our study thus touches upon is the ability of policies and strategic planning to control action, which is often assumed in public health [12, 42]. Greer and Lillvis [9] for instance emphasize plans and targets as a means to ensure implementation. Our findings question this governing optimism. Winter [16] argues that vagueness and ambiguity of policy goals are well-known in implementation research, and policy goals are not always expected or even intended to be achieved. But this does not necessarily constitute a problem. Brunsson [48] compellingly shows how inconsistent demands are a basic condition in political organizations. Elected politicians and complex government organizations purposely represent multiple conflicting interests, values and ideas. The inherent tensions between inconsistent demands should therefore not be considered an error of silo-based government, but rather an integral part of sectoral realities. Sectors purposefully represent institutionalized mobilizations of bias, and as such different interests and values [51]. By having loose couplings, i.e. solving some demands with talk, some with decision-making, and some with action, organizations are able to meet contradictory demands simultaneously [48]. Brunsson contemplates this so-called “hypocrisy” as a solution rather than a problem because no matter how positive the demands are, it is not easy – if possible at all – for an organization or a government to satisfy them all. Hence, success in one direction will often undermine success in another. Accordingly, loose couplings (and decoupling) are not necessarily dysfunctional. Rather, loose couplings between talk and actions make it possible for organizations to show support for high values by talk and decision-making, even if they are not able to act in accordance with it themselves. This way many more can support high values than would be the case if only the few who act in accordance with them were allowed to do so [48]. By adopting the strategy, Townville maintained the mandate for intersectoral action to improve health, thus maintaining and displaying good intentions that may be difficult to meet in circumstances of budget cuts and inconsistent demands. Moreover, despite not directing action, the strategy did ensure that local action was still possible, and intersectoral efforts still a formal priority in Townville, in contrast to a scenario where a political decision had been made to dismiss the strategy due to inconsistency with other demands.

While the literature on intersectoral policymaking and joined-up approaches for health is growing [3, 9, 12, 20, 50, 52], the assumption that decoupling between rhetoric and action is a failure of implementation is generally not questioned. However, with Brunsson we highlight the potential contribution of decoupling as a means to ensure flexibility that may allow for continued implementation in light of inconsistent demands, and not least the display of good intentions and thereby continued support for these values. Though a limitation of the neo-institutionalist approach is that it does not provide a prescriptive theory that can help direct practice. Rather, maintaining good intentions by decoupling requires a continued belief in the rational organization where policies or ‘talk’ control action.

### Methodology

Another contribution of our study is methodological. Shankardass et al. [20] argue convincingly to promote the methodology of the realist explanatory case study to further insights on the implementation of HiAP. In their approach, interviews are conducted 2-10 years after initiation [50]. However, we have shown how ethnography contributes with important insights about the intersectoral process, which follow-up interviews did not disclose. We argue that only relying on interview data thus limits the insights on intersectoral dynamics and thereby our understanding of the implementation of intersectoral policymaking.

A limitation of our study is that the process is not followed over a longer period of many years. Thus, we are not able to provide insights on subsequent implementation or sustainability of intersectoral efforts. Moreover, we only give detailed insight into the process in one government organization. A question for further research would be to investigate this in different contexts. Nonetheless, we have shown how the process was attributed great significance in follow-up interviews and how participants considered it a success, despite not meeting the initial intentions and not producing the expected action.

### Implications for intersectoral policymaking for health

In the Danish context, we find that Townville represents an exemplary case. Correspondingly, the inconsistent demands are somewhat similar to the contradicting logics found in Holt et al. [53]. Whereas the Danish context is unique, we find that the ‘myth’ of intersectoralism may represent more general implications for intersectoral policymaking. For instance, despite great uncertainties regarding the effects of intersectoral policymaking and reservations associated with (measuring) the outcomes [54–56], HiAP is expected to create better health and health equity, generate synergies between sectors, and deal with rising costs of the demographic development [42, 57, 58]. Moreover, health is presented as both a matter of wellbeing and happiness as well as good business: an important enabler and prerequisite for attaining both social and economic goals [42]. We find that these high values represent similar myth-like qualities, as a ‘taken-for-granted’ solution to improve health. This is in line with Carey and Crammond [46] who critique the normative bias depicting joined-up approaches, such as HiAP, as wholly positive, while being out of sync with the state of evidence. Similarly, Exworthy and Hunter [3] argue that joined-up innovations may not be the panacea often believed. Degeling [51] finds that the call for intersectoral action in public health is naïve, and goes as far as to describe intersectoral collaboration as a “contradiction in terms”. Thus we caution that intersectoral policymaking intuitively be considered a powerful solution for promoting health. Instead, further attention should be paid to how cases of successful implementation have dealt with the inherent inconsistent demands. This would make an interesting topic for further research.

## Conclusion

The paper contributes to the current literature on Health in All Policies and the broader field of policy implementation by showing how the myth of intersectoralism posed a barrier to turn the intersectoral health policy into action. Particularly three elements functioned to avoid the necessary specification that would direct action: 1) idealization of universal intersectoralism, 2) doubts about economic outcomes, and 3) tensions between inconsistent demands. By producing abstract rhetoric rather than directly addressing these challenges, the myth of intersectoral policymaking resulted in diffuse responsibility and no priorities, all the while the intuitive appeal of the myth was maintained. However, we argue that this decoupling between rhetoric and action may not simply be a failure of implementation, but also a means to sustain good intentions and hereby allow for continued action. The study thus contributes to current debates about the process and social dynamics of intersectoral policymaking for health, and particularly expands the discussion regarding policy implementation by showing how abstract ideas may result in elusive implementation.

## Additional file

Additional file 1: “General template: Interview guide for initial interviews”. The supplementary material presents the general template of the interview guide which directed the majority of the interviews. (DOCX 18 kb)

## Abbreviations

HiAP: Health in All Policies
PHO: The Public Health Office
