# Supplementary material for: Elusive implementation: an ethnographic study of intersectoral policymaking for health
Source: BMC Health Serv Res. 2018 Jan 30;18:54. doi: 10.1186/s12913-018-2864-9 (PMC5789672; doi:10.1186/s12913-018-2864-9)
Supplement: Supplementary file 1 — “General template: Interview guide for initial interviews”. The supplementary material presents the general template of the interview guide which directed the majority of the interviews. (DOCX 18 kb) [file 12913_2018_2864_MOESM1_ESM.docx]

# General template: Interview guide for initial interviews

Below is depicted a general template for the interview guides.

For the initial interviews, four general interview guides were developed for different groups of participants; top-management, head of health department, public health professionals, and partners from non-health departments. The themes in the templates were the same, while the structure as well as some specific questions varied depending on the group of participants. The four templates were further individually adjusted before each interview to accommodate participant’s specific position and organization.

For follow-up interviews, no general template was designed. The interview guides were developed specifically for each interview to enquire about the role and situation of each participant, and their experience and evaluation of the policy process, as well as their reflections and feedback to DHH’s observations and initial interpretations.

For a longer discussion and further reflections regarding the interviews, please see Holt (2016).

*General template: Interview guide (translated into English by DHH)*

| Introduction | - Briefly introduce the overall research project - Introduce the aim and themes of the interview - Inform about timeline and practicalities - Ask for consent to use the data – explaining data management and use, confidentiality etc. - Ask permission to record the conversation |
| --- | --- |
|  | Ask the participant to briefly introduce themselves in terms of position and function in the organization, and their most important tasks  *(when non-health sector participants)*   - Can you describe how you work with health promotion and prevention as part of your work? - How is health promotion and prevention important/ how does it relate to your daily operations/ daily operations of your department?   How much time/focus does health promotion and prevention take up in comparison to your other tasks? |
| Organization and collaboration | Where is ‘health’ placed in the organization diagram – who is responsible?   - Why have you chosen this organization? *(top-management only)* - What are the benefits? - What are the challenges?   What is your (department’s) role and task in terms of health promotion and prevention?   - Most important tasks? - Everyday operations (e.g. describe last week)? - What are you (your department) particularly good at? - Which challenges are you facing? - What would you deem most difficult?   Which other departments are involved in health promotion and prevention?   - Which responsibilities and tasks are placed where?   Who are you (your department) collaborating with?   - What does each partner contribute with? - What enables the collaboration? - What is difficult or challenging in terms of collaboration?   Have you experienced any conflicts relating to your role/function in the organization/ in terms of health promotion?  In terms of intersectoral organization/collaboration:   - How do you collaborate across the organization? - How are you formally organized? - What are the roles of different partners/departments? (who does what?) - How does it work in practice – explain with examples - What works well? - What is challenging/difficult? - In general, what would you deem important for a “good organization” to function?   (*Formal organizational change?)*   - *What has changed?* - *What is the main purpose and rationale of the organizational change? (what didn’t work in the former organization)* - *Can you describe the process of organizational change?* - *How is the support for the organizational changes?* - *Improvements/challenges arising from the new organization?*   Generally, what would you highlight as the greatest advantages in terms of how you are organized?  And vice versa, what are the greatest challenges you’re facing? |
| Health across | Is ‘health across’ an expression you use (you know)?   - In which situations would you use it? (give examples) - When did you last use the expression?   How would you explain health across, using your own words?   - What does it describe? - What does it entail in practice - What exactly is it that ‘cuts across’? (e.g. policies, objectives, meetings, knowledge, competencies, coordination, …etc.?) - And across what? (E.g. departments, professions, hierarchies, policies…?) - Is it possible to distinguish in terms of whether it involves a *division* of tasks between many partners and/or does it involve *shared* decision-making/ operations/…?   Why is health across important? / what does it contribute? |
| Political organization and the politicians | Which political committee(s) is/are responsible for health promotion and prevention in X municipality?   - How are political proposal/cases distributed between the political committees? - Which departments of the administration serves this (these) political committee(s) - What are the important concerns regarding this political organization?   Is it your experience that the politicians have a strong commitment in terms of health promotion and prevention?   - How is this expressed? - How do you work on establishing/ensuring political commitment? - Is health promotion and prevention a political priority? (in what way/ why not)? - Do any of the politicians hold health as a favorite cause?   Is it your experience that the politicians are knowledgeable about health promotion and prevention?   - What is generally the focus of the political discussion regarding health (health promotion and prevention)? - Generally, is it your experience that the political discussions regarding health (health promotion and prevention) are characterized by political agreement or political disagreement (examples?) |
| Economy and budgets | Is health generally a priority in the annual financial/budget negotiations?  How/where are the finances for health promotion and prevention placed in your budget (which department(s)? Integrated with bigger areas or dedicated to explicit efforts?)  Is there any funding dedicated to the implementation of your health policy?  What characterizes the main funding for health promotion and prevention in general?  (ongoing funding for operations, temporary project funding, external funding…) |
| Health policy | How do you use the health policy?   - When did you last use it/look at it? - In which situations would you use it? (give examples)   Is your health policy an intersectoral or sectoral policy?   - Who uses it? - Who is responsible for achieving the policy objectives?   Can you tell me about the process of developing your health policy?   - Who was involved? - What was the function and roles of different actors involved (individuals/departments) - What was important as part of this process? - What was challenging in this process?   In your interpretation/experience, why did you choose the specific target areas of the policy?   - What was important in the discussions? - How did you develop them? (tell about the process)   What defines a good policy/ what is important for a policy to function well?  What would you highlight as the greatest strength of your health policy?  And vice versa, what is the greatest weakness/ challenge?  How are you (the municipality) implementing the policy?   - Which actions have been taken? (give examples)   How do you evaluate the policy and its implementation?   - What kind of measures are you using? |
| Completion | Is there something you would change – in relation to how you are organized and work with health promotion and prevention – if you were free to do so?   - Something you wish you were better at?   What is the most important learning from your experiences with intersectoral collaboration/ health across?  Do you have anything further to add, maybe something important, which we haven’t touched upon in our conversation?  Thank you for participating! |
